# Confirming the Effects of Qinghuayin against Chronic Atrophic Gastritis and a Preliminary Observation of the Involved Inflammatory Signaling Pathways: An In Vivo Study

**DOI:** 10.1155/2018/4905089

**Published:** 2018-09-26

**Authors:** Sihan Li, Minghan Huang, Qin Chen, Shunan Li, Xin Wang, Jianlong Lin, Guodong Zhong, Ping Lin, Tetsuya Asakawa

**Affiliations:** ^1^Basic Medical College of Guangzhou University of Chinese Medicine, No. 232 Waihuandong Road, Guangzhou, 510006, China; ^2^Department of Gastroenterology, The Second People's Hospital Affiliated to Fujian University of Traditional Chinese Medicine, No. 282 Wusibei Road, Fuzhou, 353003, China; ^3^Department of Internal Medicine, Fujian Medical University Union Hospital, Fuzhou, China; ^4^Department of Pharmacy, The Second People's Hospital Affiliated to Fujian University of Traditional Chinese Medicine, Fuzhou, 353003, China; ^5^Department of Pathology, The Second People's Hospital Affiliated to Fujian University of Traditional Chinese Medicine, Fuzhou, 353003, China; ^6^Research Base of Traditional Chinese Medicine Syndrome, Fujian University of Traditional Chinese Medicine, Fuzhou 350122, China; ^7^Department of Neurosurgery, Hamamatsu University School of Medicine, Handayama, Hamamatsu-City, Shizuoka, Japan

## Abstract

**Background:**

Qinghuayin (QHY) is a Chinese formula that is widely used in the treatment of chronic atrophic gastritis (CAG). This study was planned with the following objectives: (1) confirming the efficacy of QHY in a rat model of CAG and (2) performing a preliminary observation of the changes in several inflammatory signaling pathways potentially involved in the QHY mechanisms.

**Methods:**

A total of 33 rats were used in this study; they were divided into the control (n = 12) and model (n = 21) groups. QHY was administrated to both the groups. We assessed the pathological manifestations and the serum tumor necrosis factor alpha (TNF-*α*) level as markers of efficacy. We also performed a preliminary observation of the changes in the protein and messenger ribonucleic acid (mRNA) expression of toll-like receptors 4 (TLR4), MyD88, NF-*κ*B, and COX-2.

**Results:**

The pathological changes induced in the rats by the establishment of the CAG models were recovered by low and high doses of QHY. Their serum TNF-*α* level also reduced following low- and high-dose QHY treatment. Protein and mRNA expressions of TLR4, MyD88, NF-*κ*B, and COX-2 were upregulated by the establishment of CAG models and downregulated by the administration of low- and high-dose QHY.

**Conclusions:**

Our data confirm the efficacy of QHY as an adjuvant therapy, based on the theories in traditional Chinese medicine. The preliminary observations indicate that the downregulation of the enhanced inflammatory signaling pathways might be crucial QHY mechanisms that need further verification.

## 1. Introduction

Chronic atrophic gastritis (CAG) is a common chronic gastric mucosal lesion that plays a crucial role in the development of gastric carcinoma, a type of cancer with a high mortality rate in China. Development of effective therapies against CAG is extremely important for reducing gastric cancer mortality. The pathogenesis of CAG is complicated, and it is well documented that* Helicobacter pylori* (HP) infection is strongly associated with CAG and the subsequent development of gastric cancer [1]. Thus, anti-HP treatment is an important therapy for CAG. Further, other therapies, such as gastric mucosa protection, symptomatic pain relief, Vitamin C supplementation, and proton pump inhibitor administration, have been used in CAG treatment [2]. However, there is no specific treatment for CAG. In China, many therapies based on the theories of traditional Chinese medicine (TCM) have been considered. A recent systemic review has suggested that a Chinese classical formula, Sijunzi decoction, may benefit CAG patients [3]. In addition to Sijunzi decoction, Qinghuayin (QHY) is another Chinese formula that is widely used in the treatment of CAG based on the TCM theories of clearing heat and resolving dampness. QHY is composed of herbs, including* semen dolichoris album* (Baibiandou)*, Poria cocos *(Fuling)*, coix seeds *(Yiyiren)*, herba artemisia scoparia* (Yinchen)*, herba eupatorii *(Peilan)*, amomum cardamom *(Baidoukou)*, rhizoma coptidis *(Huanglian)*, cortex magnolia officinalis* (Houpu), and* radix paeoniae rubra* (Chishao). Several Chinese studies have reported good efficacy/safety of QHY in CAG treatment [4–6]. However, the underlying mechanisms remain unclear.

In contrast, HP infection may affect signaling pathways [7]. CAG is also associated with inflammatory signaling pathways, such as the IL-11/STAT3 signaling pathway [8], leptin receptor signaling pathway [9, 10], E-cadherin/*β*-catenin/tcf-4 pathway [11], and PI3k/Akt pathway [12]. Wang et al. reported that Toll-like receptors 4 (TLR4) and TLR9 are regulated by HP and are closely associated to CAG [11]. We hypothesized that QHY mechanisms are also associated with the regulation of inflammatory signaling pathways. Therefore, we observed the changes in the biomarkers of several signaling pathways in rat CAG models treated with QHY. We attempted to conduct a preliminary examination of the roles played by the signaling pathways in QHY mechanisms.

## 2. Methods and Materials

### 2.1. Animals

A total of 33 male Wistar rats (body weight 110 g ± 10 g) were involved in the trial. All the rats were carefully treated as per the National Institute of Health Guidelines for the Care and Use of Laboratory Animals. All the experiments were approved and supervised by the Animal Care and Use Committee of the Fujian University of Traditional Chinese Medicine (approval number: 3100101037). The rats were raised in a room at 23°C with normal rhythm (8:00 AM to 8:00 PM, light on).

### 2.2. QHY Preparation

The QHY compound was prepared according to the formula approved by the Fujian Provincial Food and Drug Administration (approval number: Z05104030). The ingredients of the QHY solution have been listed in Table 1. We filtered the decoction and adjusted the concentration to 2.2 g/mL (raw materials) at 60°C. The solution was bottled and sterilized for the experiments.

### 2.3. Experimental Design

The rats were raised normally until 1 week before the experiments; thereafter, they were randomly divided into the control group (C, n = 12) and the CAG model group (M, n = 21). The control group rats were raised with normal water and diet. Rats in the model group were allowed free access to 0.5 g/L ammonia as drinking water during the initial 12 weeks. Then, they were gavaged once daily with 20 mmol/L sodium deoxycholate solution (2 mL each time) and gavaged with 60% ethanol two times a week (2 mL each time). At the end of 12 weeks, 3 rats from the model group were selected randomly and deeply anesthetized with chloral hydrate (400 mg/kg). The stomach was removed for pathological examination to confirm the successful establishment of the CAG model.

QHY doses were defined as per the dose in human patients: 1.5 mL/kg was defined as low-dose treatment (LT), while 6.0 mL/kg was considered high-dose treatment (HT). All the agents were diluted with saline to make up a 4-mL solution for gavage.

The control group rats were randomly divided into the following two groups: Intact group (n = 6) underwent gavage with 4 mL saline; the C + HT group (n = 6) underwent gavage with high-dose QHY (6.0 mL/kg). The rat CAG models were randomly divided into the following 3 groups: M group (n = 6) that underwent gavage with 4 mL saline, the M + LT group (n = 6) that underwent gavage with low-dose QHY (1.5 mL/kg), and the M + HT group (n = 6) that underwent gavage with high-dose QHY (6.0 mL/kg).

Gavage was performed continuously for a period of 30 days. On the 31st day, after blood sampling (3 mL) for the measurement of the serum TNF-*α* level, animals were sacrificed with an intraperitoneal injection of chloral hydrate (400 mg/kg) and their stomachs were removed for pathological examination.

### 2.4. Serum Tumor Necrosis Factor -*α* (TNF-*α*) Concentrations

The serum TNF-*α* level was measured using a standard enzyme-linked immunosorbent assay (ELISA) method. The rat blood samples (3 mL) were subjected to low-temperature freezing and centrifuged at 3500 rpm at 4°C for 15 min; the supernatant was collected, and measurement steps were conducted as per the manual of the ELISA kit (Shanghai Westang Bio-Tech. Co. LTD, China).

### 2.5. Pathological Examination

Each stomach sample was divided into two parts as follows: two blocks of tissues (the same size as soybeans) were clipped at the gastric antrum and immediately placed in liquid nitrogen for real-time polymerase chain reaction (RT-PCR) and western blot analysis; the remaining gastric antrum tissues were used for the hematoxylin and eosin (HE) staining.

#### 2.5.1. HE Staining

Tissues were fixed with 4% paraformaldehyde for 24 h; thereafter, the fixed-conventional paraffin embedded tissues were cut into 5 consecutive slices (slice thickness 4 *μ*m), HE staining was performed, and histopathological changes in the gastric mucosa were observed under an optical microscope.

### 2.6. Detection of the mRNA Expression of TLR4, MyD88, NF-*κ*B, and COX-2

A standard RT-PCR was employed for detecting the mRNA expression of TLR4, MyD88, NF-*κ*B, and COX-2. Briefly, after total RNA extraction, RT-PCR was performed in the following conditions using a quantitative real-time RT-PCR machine (Applied Biosystems, 7900 Fluorescence-based quantitative PCR system, Carlsbad, USA): predenaturation at 50°C for 2 min and denaturation at 95°C for 10 min; 95°C for 15 s and 60°C for 1 min, with 40 cycles of amplification. The RNA level was measured with a ND2000C Ultra-micro spectrophotometer (Thermo ScienTific, USA). With the Ct value as the target gene expression and GAPDH as the internal control, the relative expression of target gene was calculated by 2^−ΔΔCt^.

Primers were as follows: TLR4 sense primer, 5'-GCCCTCAGTCTTGGAGTGTC-3'; TLR4 antisense primer, 5'-TAACACAGGGCGCCTAAGAG-3'; product length, 93bp; NF-*κ*B sense primer, 5'-AGTTTGACGGTGAGCTGGTA-3'; NF-*κ*B antisense primer, 5'-GCCTCGGCCTGCCGCAAGCCT-3'; product length, 300bp; COX-2 sense primer, 5' -GGC TGTATATCTGCTCTATATGC-3'; COX-2 antisense primer, 5' -CCGCTTCCTTTGTCCATCAG-3'; product length, 306bp; MyD88 sense primer, 5' -GGACTGCCAGAAATACATACGC-3'; MyD88 antisense primer, 5' -CTTGTCTGTGGGACACTGCTC-3'; product length, 94bp; GAPDH sense primer, 5'-CAACGGGAAACCCATCACCA-3'; GAPDH antisense primer, 5' -ACGCCAGTAGACTCCACGACAT-3'; product length, 96bp;

### 2.7. Detection of the Protein Expression of TLR4, MyD88, NF-*κ*B, and COX-2

A standard western blot analysis was used for detecting the mRNA expressions of TLR4, MyD88, NF-*κ*B, and COX-2. Protein expressions were measured using a DCTM protein assay kit (Bio-Rad, USA). Briefly, gastric antrum tissues (150 mg) were lysed for total protein extraction; protein samples (30 *μ*g) were subjected to SDS-PAGE electrophoresis (Bio-Rad, USA) and then transferred to a nitrocellulose membrane and blocked with TBST for 1 h; primary antibodies including TLR4 (1:800), MyD88 (1:500), NF-*κ*B (1:1000), COX-2 (1:800), and *β*-actin (1:1000) were used for overnight incubation at 4°C. Subsequently, the membranes were washed thrice with TBST, followed by incubation with second antibodies for 1 h. Enhanced chemical fluorescein imaging and photographing were performed. Image analysis was performed using Graphic analysis software (Ascent software for Multiskan, Thermo ScienTific, USA), and the ratio of the gray values of the target bands to the internal reference (*β*-actin) was calculated.

### 2.8. Statistical Analyses

Data were analyzed using SPSS 19.0.0 software (SPSS Inc., IL, USA). The data are presented as mean ± standard deviation values. One-way analysis of variance was performed followed by a Dunnett post hoc test for multiple comparisons. Categorical data were analyzed using the rank sum test. P < 0.05 was considered statistically significant.

## 3. Results

### 3.1. Confirming the Efficacy of QHY

#### 3.1.1. Evidence of Pathological Examination

We confirmed the pathological changes in both groups. In the control group, we observed normal pathological manifestation in the gastric mucosa. Gastric mucosal epithelial cells and glands were consistent in shape and size; they were neatly and closely arranged. The cells were single-layer columnar, and the boundary between the glandular epithelium and the glandular tube was clear; no hyperplasia was observed. No dilation or blood stasis was observed in the inherent gland and mucosa, and there was no infiltration of the inflammatory cells, intestinal metaplasia, or obvious abnormalities in the stroma and muscularis mucosa. QHY treatment did not change the pathological manifestation in the control rats.

In contrast, in the CAG models, we observed thinning of the gastric mucosa, reduction of the glands, cystic dilation in the individual glands accompanied by varying degrees of intestinal epithelial metaplasia and dysplasia, thickened muscularis mucosa, and cell infiltration (interstitial lymphocytes and plasma cells) with partial patchy lymphocyte aggregation lesions. The pathological manifestation confirmed successful establishment of the CAG rat models.

**Figure 1:**
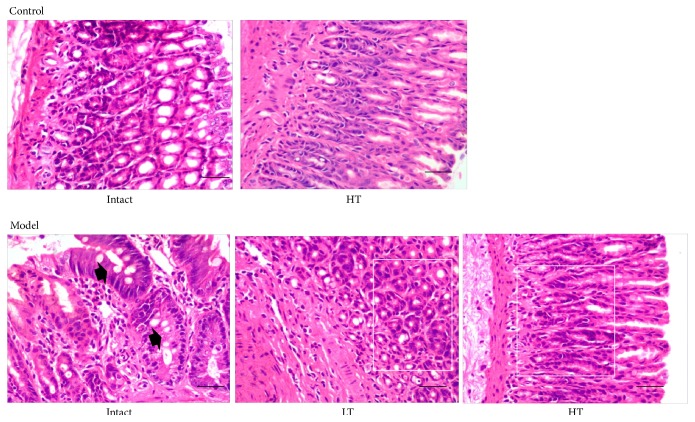
Representative images of the gastric mucosa in the different groups of rats. In the control group, the rats who underwent no treatment (Intact) and those who were administered high-dose QHY treatment (HT) exhibited normal gastric mucosa. Their glands were shaped normally, and there was no atrophy of the gastric glands or intestinal metaplasia. In the model group rats that were not given any treatment (Intact), there was obvious atrophy of the gastric glands and intestinal metaplasia (arrows). However, the group that underwent treatment with low-dose QHY (LT) and high-dose QHY (HT) showed improvement in the pathological manifestations. No intestinal metaplasia was observed, and the gastric glands were thicker than those of the model group (areas in the white frame). Black bar = 1,000 *μ*m.

Treatment with high- and low-dose QHY may ameliorate these variations. We observed thicker gastric mucosa, more normal glands, and less cell infiltration (Figure 1). The pathological changes also confirmed the efficacy of QHY.

#### 3.1.2. Reduction in the TNF- *α* Level Following QHY Administration

**Figure 2:**
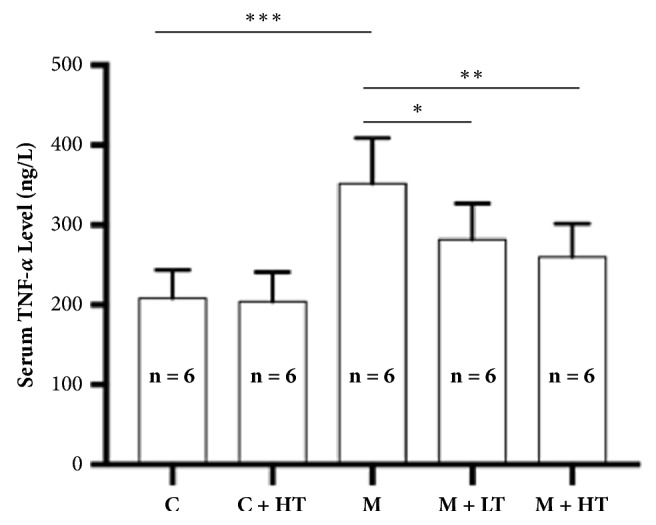
Serum TNF-*α* levels of the different groups of rats. The serum TNF-*α* level of the rats in the model group (M) was significantly higher than that of those in the other groups. Treatment with low-dose QHY (M + LT) significantly reduced the increase in serum TNF-*α* levels (p < 0.05); in contrast, high-dose treatment of QHY (M + HT) demonstrated superior efficacy (p < 0.01). All the values represent mean ± standard deviation (SD); ^*∗*^means P < 0.05, ^*∗∗*^means P < 0.01, and ^*∗∗∗*^means P < 0.001. HT = high-dose QHY treatment, LT = low-dose QHY treatment, and M = model mice without treatment.

We found that the serum TNF-*α* level was significantly enhanced in the CAG model rats (p < 0.001). Treatment with low- and high-dose QHY significantly reduced the serum TNF-*α* level. High-dose QHY had a significantly higher efficacy than low-dose QHY (p < 0.05). Reduction in the serum inflammatory factors may be a comprehensive result of the inhibition of several inflammatory signaling pathways (Figure 2).

Based on the pathological examinations and the serum TNF-*α* level, we confirmed the efficacy of QHY as an adjuvant therapy for CAG in a rat model.

### 3.2. Preliminary Observations of the Effects of QHY on Several Inflammatory Signaling Pathways

In this study, we conducted a preliminary observation of the mRNA expression and protein expression of the TLR4, MyD88, NF-*κ*B, and COX-2 signaling pathways.

**Figure 3:**
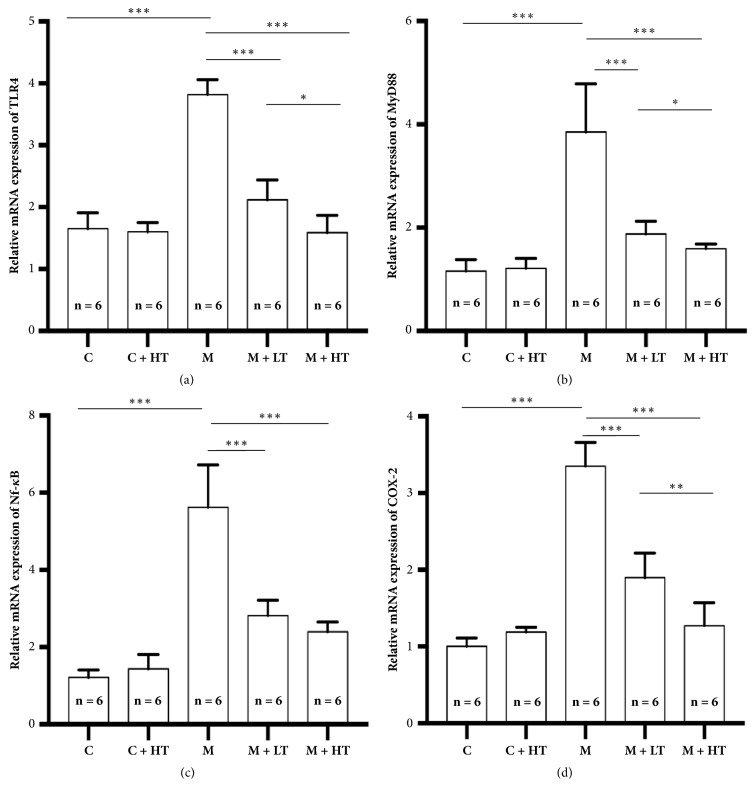
mRNA expression of TLR4, MyD88, NF-*κ*B, and COX-2 in the different groups of rats. (a) The mRNA expression of the TLR4 signaling pathway. The mRNA expression of TLR4 in the model group (M) was significantly higher than that of those in the other groups. Treatment with low-dose QHY (M + LT) significantly downregulated the enhancement of the mRNA expression of TLR4 (p < 0.001); in contrast, treatment with high-dose QHY (M + HT) achieved significantly more reduction (p < 0.001). The high-dose and low-dose exerted significantly different effects (p < 0.05). (b) The mRNA expression of the MyD88 signaling pathway. (c) The mRNA expression of the NF-*κ*B signaling pathway. (d) The mRNA expression of the COX-2 signaling pathway achieved results similar to those of TLR4 only in the NF-*κ*B signaling pathway; there was no significant difference between the high- and low-dose QHY treatments. All the values represent mean ± standard deviation (SD); ^*∗*^means P < 0.05, ^*∗∗*^means P < 0.01, and ^*∗∗∗*^means P < 0.001. HT = high-dose QHY treatment, LT = low-dose QHY treatment, and M = model mice without treatment.

Our results showed that the mRNA expression of the TLR4, MyD88, NF-*κ*B, and COX-2 signaling pathways was upregulated in the rat models with CAG and can be significantly downregulated by treatment with high- and low-dose QHY. Furthermore, high-dose QHY exhibited a stronger efficacy for the TLR4, MyD88, and COX-2 pathways (Figures 3(a), 3(b), and 3(d)).

**Figure 4:**
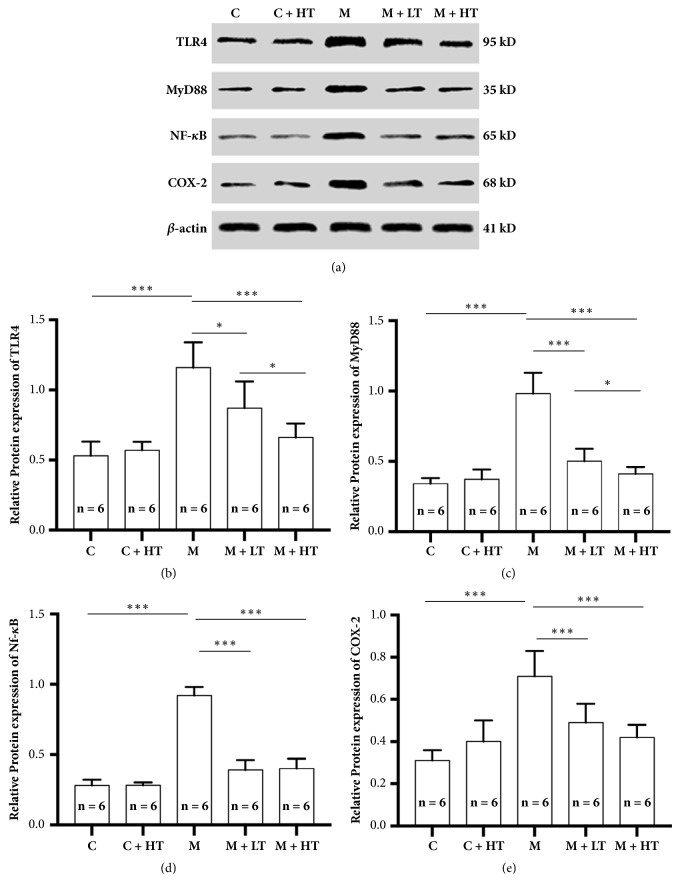
The protein expression of TLR4, MyD88, NF-*κ*B, and COX-2 in different groups. (a) Representative images of western-blot analyses. (b) The protein expression of the TLR4 signaling pathway. The protein expression of TLR4 in the model group (M) was significantly higher than those in the other groups. Treatment with low-dose QHY (M + LT) significantly downregulated the enhancement of the protein expression of TLR4 (p < 0.05); in contrast, treatment with high-dose QHY (M + HT) achieved significantly more downregulation (p < 0.001). The difference between the effects of high-dose and-low dose QHY was significant (p < 0.05). (c) The protein expression of the MyD88 signaling pathway. The protein expression of MyD88 in the model group (M) was significantly higher than that of those in the other groups. Treatment with low-dose QHY (M + LT) significantly reduced the enhancement of MyD88 protein expression (p < 0.001); however, treatment with high-dose QHY (M + HT) achieved a more significant reduction (p < 0.001). The difference between the effect of high-dose and low-dose was significant (p < 0.05). (d) The protein expression of the NF-*κ*B signaling pathway. The protein expression of NF-*κ*B in the model group (M) was significantly higher than that of those in the other groups. Treatment with low-dose QHY (M + LT) and high-dose QHY (M + HT) caused a significant downregulation in the enhancement of the protein expression of NF-*κ*B (p < 0.001). There was no significant difference in the effects of high-dose and low-dose treatment. (e) The protein expression of the COX-2 signaling pathway. The protein expression of COX-2 in the model group (M) was significantly higher than those in the other groups. Treatment with low-dose QHY (M + LT) and high-dose QHY (M + HT) significantly downregulated the enhancement COX-2 protein expression (p < 0.001). No significant difference was observed between the effects of high-dose and low-dose treatment. All the values represent mean ± standard deviation (SD); *β*-actin was used as the internal control. ^*∗*^means P < 0.05, ^*∗∗*^means P < 0.01, and ^*∗∗∗*^means P < 0.001. HT = high-dose QHY treatment, LT = low-dose QHY treatment, and M = model mice without treatment.

The protein expression of the TLR4, MyD88, NF-*κ*B, and COX-2 signaling pathways was enhanced in the rat models with CAG. High- and low-dose QHY treatment can downregulate such enhancements in the protein expression of signaling pathways associated with CAG. However, only in the TLR4 and MyD88 pathways, high-dose QHY treatment exhibited a strong efficacy for downregulation (Figures 4(a) and 4(b)).

These results indicate that the downregulation of the enhanced inflammatory signaling pathways play a crucial role in QHY mechanisms.

## 4. Discussion

In this study, we confirmed the efficacy of QHY by examining the pathological slices and serum TNF-*α* level in CAG rat models. We also performed preliminary observations to determine the effects of QHY on several inflammatory signaling pathways. Our results suggest that QHY is an efficient treatment for CAG. Our data also indicate that downregulation of the enhanced inflammatory signaling pathways plays a crucial role in the QHY mechanisms that need further verification. To the best of our knowledge, this is the first to investigate the efficacy of QHY in CAG treatment, and the results of this study will contribute to CAG treatment with alternative therapies, including medicines based on TCM.

Pathological examination showed us that pathological changes that occur after the administration of sodium deoxycholate solution were recovered with both low-dose and high-dose QHY treatment. Typical pathological changes of CAG, such as atrophy of the gastric glands and intestinal metaplasia, were clearly observed in the intact model group, indicating successful establishment of the rat models of CAG. It is noteworthy that QHY administration can ameliorate these pathological changes. In our samples, intestinal metaplasia disappeared and the gastric glands became thicker in the rat groups administered high- and low-dose QHY treatment. Moreover, the pathological manifestations remained unchanged in the control group rats that were given QHY treatment. This result may indicate good safety of QHY; this result warrants verification (Figure 1).

In contrast, the serum TNF-*α* level is considered to be a sensitive biomarker of HP infection and CAG [13]. Therefore, we further verified this by measuring the serum TNF-*α* level. Our data indicate that the serum TNF-*α* level of the model group rats was significantly higher than that of the control group rats. The administration of high- and low-dose QHY treatment can significantly reduce the serum TNF-*α* level, although the levels did not reach the normal range; there was no significant difference between the effects of high- and low-dose QHY treatment. These data further proved the successful establishment of rat models of CAG and the efficacy of QHY (Figure 2).

With respect to the mechanisms, we performed preliminary observations for the mRNA and protein expressions of TLR4, MyD88, NF-*κ*B, and COX-2 after QHY administration. All the results were analogous; the expressions of TLR4, MyD88, NF-*κ*B, and COX-2 were upregulated after the administration of sodium deoxycholate solution (establishment of the rat CAG model). QHY administration could significantly downregulate the expressions of TLR4, MyD88, NF-*κ*B, and COX-2, while high-dose QHY exhibited better effects (Figures 3 and 4). These results are in accord with previous results [7, 8] that indicate that the downregulation of the expressions of several inflammatory signaling pathways, such as TLR4, MyD88, NF-*κ*B, and COX-2, plays a crucial role in QHY mechanisms. Several inflammatory signaling pathways are involved in the QHY mechanisms, and we believe that these mechanisms are complicated. We are unaware of the number of inflammatory signaling pathways involved in the mechanisms as well as the strength and interactions of these pathways.

In contrast, some reports from China suggest that QHY inhibits HP [14, 15]. In fact, some ingredients of QHY (Table 1) have the ability to inhibit HP activity. Several studies have reported that* rhizoma coptidis extract *inhibits the urease of HP [16–19]. Epiberberine is the lead urease inhibitor that may contribute to the treatment of HP-induced CAG [17]. Cortex magnolia officinalis is another herb that has been proven to have anti-HP activity [18, 20]. The activation of the inflammatory signaling pathways plays a crucial role in the pathogenicity of HP [21]. The inhibition of HP and the suppression of the HP-related inflammatory signaling pathways might be important underlying mechanisms for QHY efficacy. These potential biological interactions between HP and QHY will be further investigated in our next study.

The present study is based on experiments conducted on a rat model. Although it demonstrated changes in several inflammatory signaling pathways induced by QHY, we are aware that the results from animal models sometimes are not exactly similar to those in human subjects. In order to clarify the QHY mechanisms, clinical studies on human subjects, along the lines of those conducted on the rat model, are crucial.

In our future study, we will confirm each inflammatory signaling pathway using an agonist or antagonist of that particular pathway. We shall attempt to find more inflammatory signaling pathways that are involved in the QHY mechanism and understand the QHY mechanisms. The potential biological interaction between HP and QHY warrants further investigation. Rigorous pharmacological component analysis of QHY and investigation in human patients are crucial for the further application of QHY as an adjuvant therapy for CAG.

## 5. Conclusions

In sum, the present study confirmed the efficacy of QHY as a TCM on the basis of pathological examination and serum TNF-*α* level in a rat model. Our preliminary observation of the inflammatory signaling pathways demonstrated the involvement of several pathways, such as TLR4, MyD88, NF-*κ*B, and COX-2, in the effects exerted by QHY. The downregulation of the enhanced inflammatory signaling pathways might be a crucial underlying mechanism for the effects of QHY. Although the present study confirmed the efficacy of QHY as an adjuvant therapy against CAG, the mechanisms underlying QHY require further exploration.

## Figures and Tables

**Table 1:** Ingredients of the QHY solution.

Ping yin	English name	Proportion of ingredients
Baibiandou	*semen dolichoris album*	20.408%
Fuling	*Poria cocos*	20.408%
Yiyiren	*coix seeds*	20.408%
Yinchen	*herba artemisia scoparia*	10.204%
Peilan	*herba eupatorii*	6.123%
Baidoukou	*amomum cardamom*	3.061%
Huanglian	*rhizoma coptidis*	3.061%
Houpu	*cortex magnolia officinalis*	6.123%
Chishao	*radix paeoniae rubra*	10.204%

## Data Availability

The data used to support the findings of this study are included within the article.
